# Pattern Identification in Patients with Functional Dyspepsia Using Brain–Body Bio-Signals: Protocol of a Clinical Trial for AI Algorithm Development

**DOI:** 10.3390/jcm14041072

**Published:** 2025-02-07

**Authors:** Won-Joon Koh, Junsuk Kim, Younbyoung Chae, In-Seon Lee, Seok-Jae Ko

**Affiliations:** 1 Department of Korean Medicine, Graduate School, Kyung Hee University, Seoul 02453, Republic of Korea; sambong0130@gmail.com; 2 School of Information Convergence, Kwangwoon University, Seoul 01897, Republic of Korea; junsuk.kim@office.kw.ac.kr; 3 Department of Meridians and Acupoints, College of Korean Medicine, Kyung Hee University, Seoul 02453, Republic of Korea; ybchae@khu.ac.kr; 4 Department of Digestive Diseases, College of Korean Medicine, Kyung Hee University, Seoul 02453, Republic of Korea; 5 Department of Korean Internal Medicine, College of Korean Medicine, Kyung Hee University, Kyung Hee University Hospital at Gangdong, Seoul 05278, Republic of Korea

**Keywords:** functional dyspepsia, Korean medicine, artificial intelligence, pulse diagnosis, brain–body interaction, bio-signals

## Abstract

**Background:** Functional dyspepsia (FD) is a common functional gastrointestinal disorder characterized by chronic digestive symptoms without identifiable structural abnormalities. FD affects approximately 8–46% of the population, leading to significant socioeconomic burdens due to reduced quality of life and productivity. Traditional medicine utilizes differential diagnosis through comprehensive examinations, which include observing and questioning, abdominal examination, and pulse diagnosis for functional gastrointestinal disorders. However, challenges persist in the standardization and objectivity of diagnostic protocols. **Methods:** This study aims to develop an artificial intelligence-based algorithm to predict identified patterns in patients with functional dyspepsia by integrating brain–body bio-signals, including brain activity measured by functional near-infrared spectroscopy, pulse wave, skin conductance response, and electrocardiography. We will conduct an observational cross-sectional study comprising 100 patients diagnosed according to the Rome IV criteria, collecting bio-signal data alongside differential diagnoses performed by licensed Korean medicine doctors. The study protocol was reviewed and approved by the Institutional Review Board of Kyung Hee University Hospital at Gangdong on 25 January 2024 (IRB no. KHNMCOH 2023-12-003-003) and was registered in the Korean Clinical Trial Registry (KCT0009275). **Results:** By creating AI algorithms based on bio-signals and integrating them into clinical practice, the objectivity and reliability of traditional diagnostics are expected to be enhanced. **Conclusions:** The integration of bio-signal analysis into the diagnostic process for patients with FD will improve clinical practices and support the broader acceptance of traditional-medicine diagnostic processes in healthcare.

## 1. Introduction

Diagnosis and prognosis are critical parts of the medical process, with treatment and follow-up management strategies based on the diagnosis of a disease. In traditional medicine, such as traditional Chinese and Korean medicine, instead of identifying the disease itself, a pattern of symptoms is identified based on overall health conditions, including disease-related symptoms (e.g., gastrointestinal symptoms) and other lifestyle factors (e.g., working hours, sleep patterns, constitution, etc.) and subjective experiences that may not be detected by diagnostic medical devices (e.g., endoscopy, blood tests). This approach is known as the pattern identification method [1]. Based on the identified pattern, Korean medicine doctors prescribe herbal medicine, acupuncture, and manual therapies such as Chuna. Generally, Korean medicine is used primarily for musculoskeletal disorders, functional gastrointestinal disorders, and psychological conditions. In Korean medicine, functional dyspepsia (FD) is diagnosed using a technique involving four phases of examination: interview and observation, listening and smelling, questioning, and palpation. However, key diagnostic methods such as tongue diagnosis, pulse diagnosis, and abdominal diagnosis remain subjective and lack standardization. Recent advancements have included the development of standardized questionnaires to facilitate quantitative assessments for patterns, such as spleen and stomach deficiency–cold pattern, spleen and stomach damp–heat pattern, and food stagnation pattern in patients with FD [2]; However, diagnostic protocols used in clinics still heavily rely on these four examinations. Pulse diagnosis is a cornerstone in traditional medicine for determining conditions such as deficiency–excess (deficient or excessive energy level) and cold–heat patterns. For this examination, doctors continue to rely on their tactile perception, and various medical subdisciplines propose different methods for palpation and interpretation of these subjective sensations, leading to variability and challenges in objective assessments [3,4]. FD poses a significant challenge in healthcare due to its chronic nature and the lack of standardized, objective diagnostic tools. The diagnostic process remains largely reliant on the practitioner’s expertise and interpretation, leading to variability in diagnosis and treatment outcomes. Additionally, there is a critical gap in integrating objective, quantifiable data into the diagnostic process to enhance accuracy and consistency in the diagnosis of FD. Thus, a new approach is needed to address this gap by developing more comprehensive and data-driven diagnostic models.

Pulse wave signals, generated by radial artery pulsations, reflect hemodynamic changes. Technological advancements have led to the development of pulse diagnostic devices that analyze variables, such as power spectrum, signal amplitude, frequency, regularity, and volume. In particular, the measurement of pulse signals and the analysis of pulse wave signals are increasingly used for the diagnosis and monitoring of cardiovascular and systemic diseases like diabetes, thereby expanding their clinical applications. Recent studies have explored artificial intelligence (AI) techniques to analyze pulse wave time-series data, aiming to extract clinically meaningful information and provide objective interpretations of traditional pulse patterns with significant prediction accuracy [5,6,7,8].

Functional dyspepsia (FD) is a relapsing and remitting chronic gastrointestinal disorder characterized by postprandial fullness, early satiation, and epigastric pain or burning not associated with defecation, which are not fully explained by other medical conditions [9]. Patients with FD exhibit altered brain activity in regions including the prefrontal cortex, somatosensory cortex, insula, anterior cingulate cortex, amygdala, and hippocampus [10,11,12]. Additionally, autonomic nervous system activity is an important diagnostic factor in gastrointestinal disorders, as imbalanced sympatho-vagal and autonomic responses are associated with alterations in gastrointestinal functions and symptoms [13]. For example, patients with FD showed a significantly increased skin conductance response (SCR) in response to external stressful stimuli compared to healthy controls [14], and patients diagnosed with both postprandial distress syndrome and epigastric pain syndrome showed a significantly greater low frequency/high frequency ratio measured by heart rate variability (HRV) after a drink test [15].

Although the single measurement of bio-signals (e.g., ECG, SCR, pulse, and functional near-infrared spectroscopy [fNIRS]) has contributed to the scientific understanding of the underlying pathophysiological status of FD, integrating multiple bio-signals may provide a more comprehensive understanding of FD and enhance the objectivity of diagnostic outcomes. This is because brain–body interactions and patterns in multivariate data may reveal crucial patterns useful for the diagnosis of FD. For example, the integration of brain–body bio-signals (multimodal measurements of neuroimaging data and autonomic metrics) with AI algorithms has shown the potential to address the subjectivity of pain reports [16,17], which may lead to the development of precision pain treatments [18]. However, until now, such an approach has not been attempted for pattern differentiation diagnosis of FD. A few studies have employed machine learning techniques to predict treatment responses [19,20] and identify key clinical symptoms in FD [21]. However, no studies have developed AI models using bio-signal data for pattern identification of FD. Since the diagnosis of FD is primarily conducted subjectively, this approach is not only novel but also clinically valuable. By leveraging advanced AI techniques to analyze these bio-signals, our research bridges the gap between traditional diagnostic practices and modern technological advancements, providing a more comprehensive understanding of patient conditions and potentially improving treatment outcomes.

We hypothesize that incorporating objective bio-signal data obtained from the brain and the body will enhance the predictive accuracy of differential diagnoses in patients with FD. This study aims to bridge the gap by developing an AI-based algorithm that integrates brain–body bio-signals to predict pattern differentiation outcomes, potentially contributing to the standardization and objectification of diagnostic methods in Korean medicine. To achieve this goal, we will conduct a cross-sectional clinical trial with 100 patients diagnosed with FD by ROME IV criteria, measuring brain activities, autonomic metrics, and pulse signals.

## 2. Methods

### 2.1. Study Design and Setting

This is an observational cross-sectional study combined with the development of an AI model. It will be conducted at Kyung Hee University Hospital in Gangdong, South Korea. Table 1 provides a summary of the study flowchart.

### 2.2. Ethical Considerations

The study protocol was reviewed and approved by the Institutional Review Board of Kyung Hee University Hospital at Gangdong on 25 January 2024 (IRB no. KHNMCOH 2023-12-003-003). This study was registered in the Korean Clinical Trial Registry, named Clinical Research Information Service (CRIS, registration no. KCT0009275). All information regarding the study protocol will be provided to each participant. Written informed consent will also be obtained. The study will adhere to the Declaration of Helsinki and Good Clinical Practice guidelines. Participants have the right to withdraw from the study at any time. If a participant withdraws consent or does not comply with study procedures, their participation may be terminated. Participants will also be withdrawn if they are found to violate inclusion criteria or if any adverse events or comorbidities occur that make continued participation inappropriate.

### 2.3. Sample Size Calculation

Based on machine learning model requirements for predictive validity with categorical variables, a minimum of 50 samples per category is deemed necessary [22,23,24,25]. Considering potential variability and ensuring sufficient data for model training and validation, we aim to recruit 100 patients with FD.

### 2.4. Eligibility Criteria

#### 2.4.1. Inclusion Criteria

The study will include adults aged between 19 and 65 years who have undergone gastrointestinal endoscopy, have not been diagnosed with other gastrointestinal diseases, and have been diagnosed with FD according to the Rome IV criteria. Participants must have experienced persistent symptoms for a minimum duration of three months, with the onset of these symptoms occurring at least six months prior to the study. Additionally, all participants will need to provide written informed consent before their inclusion in the study.

#### 2.4.2. Exclusion Criteria

The exclusion criteria for the study are as follows: participants with gastrointestinal structural diseases diagnosed via endoscopy within the past three years and individuals exhibiting alarm symptoms, such as significant weight loss, hematochezia, or dysphagia. The study will also exclude individuals with major psychiatric disorders or neuropsychiatric conditions and serious organic diseases that could affect bio-signals, including heart failure, angina, myocardial infarction, arrhythmia, or valvular heart disease. The use of medications that could influence bio-signals on the day of measurement, such as anticoagulants, vasodilators, and antihypertensives, will be another exclusion criterion. Physical conditions that would impede the attachment of biometric sensors, such as skin disorders at the sites where electrodes will be attached (e.g., wrist), will also result in exclusion. Participants who have undergone gastrointestinal surgery within the past six months and pregnant women will not be included. Additionally, individuals affiliated with the research team, either as students or staff, will not be eligible to participate. Finally, any potential participant deemed unsuitable by the principal investigator due to physical or mental incompatibilities will be excluded from the study during the phone screening and screening visit.

### 2.5. Participant Recruitment

Participants will be recruited through online postings and offline posters at Kyung Hee University Hospital in Gangdong. Flyers will also be sent to patients with FD who participated in previous studies by the same research team and who have consented to be contacted for participation in other studies. Recruitment materials will include detailed information about the study’s purpose, procedures, duration, compensation, and safety considerations.

### 2.6. Study Procedures

The study is organized into two in-person visits. The initial phone screening will be conducted to gather information on recent upper endoscopy and blood test results, symptoms and diagnosis of functional dyspepsia, medical history, medication use, alarm symptoms, and the inclusion and exclusion criteria. Participants deemed eligible for study participation during the phone screening will be invited to attend the first visit, which is dedicated to screening the participants, while the second visit will focus on bio-signal measurements.

During the first visit, participants will be provided with a detailed explanation of the study, and written informed consent will be obtained. Demographic data, medical and surgical history, and current medication use will be collected. Vital signs, including blood pressure, heart rate, and body temperature, will be measured, alongside anthropometric data, such as height and weight. Blood tests and an electrocardiogram (ECG) will be conducted unless results from the past year are available. FD symptoms will be assessed using the Visual Analog Scale (VAS), and a diagnosis will be confirmed through the Rome IV criteria. Eligibility will be determined based on the aforementioned inclusion and exclusion criteria.

The second visit will involve a review of medication use and menstrual cycle details for female participants. The vital signs will be measured again, and participants will complete a series of questionnaires, including the Standard Tool for Pattern Identification of FD [2], Cold–Heat Pattern Questionnaire [26], Deficiency–Excess Pattern Questionnaire [27,28], Phlegm Pattern Questionnaire [29], Food Retention Questionnaire [30], Spleen Qi Deficiency Questionnaire [31,32], Stomach Qi Deficiency Questionnaire [33], Nepean Dyspepsia Index (NDI) [34], State-Trait Anxiety Inventory (STAI) [35], Beck Depression Inventory-II (BDI-II) [36], and the EeroQol-5-Dimension (EQ-5D) [37]. Participants may opt out of answering any questions they find uncomfortable. The Standard Tool for Pattern Identification of FD questionnaire comprises 45 items that categorize FD symptoms into six types: liver–stomach disharmony, dampness and heat in the spleen and stomach, spleen and stomach deficiency and cold, tangled cold and heat, food retention disorder, and insufficiency of stomach yin. This questionnaire demonstrates high reliability, indicated by its internal consistency, and a moderate level of validity, based on correlation analyses between the FD patterns identified by the tool, the Rome IV subtypes, and other dyspepsia-related questionnaires [2]. In addition, the Cold–Heat Pattern Questionnaire [26], Deficiency–Excess Pattern Questionnaire [27,28], Phlegm Pattern Questionnaire [29], Food Retention Questionnaire [30], Spleen Qi Deficiency Questionnaire [31,32], and Stomach Qi Deficiency Questionnaire [33] have been developed as pattern identification tools. These patterns are recognized as being significantly related to FD and the tools have been validated for both reliability and validity.

### 2.7. Data Collection

Pattern identification will be conducted by licensed Korean medicine doctors via questioning, tongue diagnosis, pulse diagnosis, and abdominal diagnosis. This assessment determines deficiency–excess, cold–heat, and the presence of phlegm or food stagnation, as well as six primary FD differentiation patterns (spleen and stomach deficiency–cold, spleen Qi deficiency, liver–stomach disharmony, food stagnation, spleen deficiency with damp–heat, mixed cold and heat).

Bio-signal measurements will be conducted during the second visit or during the first visit only if the screening procedures confirm that the participants meet the inclusion criteria. Prefrontal cortex activity will be measured using a headband-type fNIRS device (NIRSIT; OBELAB Inc., Seoul, Republic of Korea). SCR, ECG, and pulse wave signals will be recorded with electrodes attached to the fingers, wrist, and chest, connected to the Biopac MP160 system (BIOPAC Systems Inc., Goleta, CA, USA). Radial artery pulse waves will also be measured using the DMP-LIFE PLUS pulse analyzer (DAEYOMEDI Co., Ltd., Ansan, Republic of Korea). These measurements will be conducted with the participants in supine position for two minutes. To maintain objectivity, the researcher measuring biometric signals will be different from the doctors diagnosing the patients with FD. Additionally, the diagnosis and pattern identification results will be blinded to both the researcher and the participants. Throughout the measurements, participants will be monitored thoroughly for any discomfort or adverse events.

### 2.8. Statistical Analysis

Categorical variables include pattern identification results diagnosed by the Korean medicine doctors and those obtained from questionnaires for pattern identification. These variables will be used for subgroup analyses and as outcomes for AI-based prediction models, facilitating comparisons of differentiation patterns based on specific diagnostic results and bio-signals.

The questionnaire results of the NDI, STAI, BDI-II, and EQ-5D will be treated as numerical variables. The normality of the questionnaire results will be assessed to determine suitable statistical tests. Depending on normality, statistical tests like *t*-tests, analysis of variance, or non-parametric tests will be used to compare groups. Subgroup analyses will compare bio-signals between different diagnostic patterns. Correlation analyses will explore the relationships between the questionnaire results and categorical variables, while mediation and moderation analyses will assess potential factors that mediate or moderate the effects among the variables.

Bio-signal data, including fNIRS, SCR, ECG, and pulse waves, will be processed as both numerical and time-series data. First, basic preprocessing steps will be applied to the bio-signal data. For fNIRS data, preprocessing will be conducted using NIRSIT QUEST software (NIRSIT; OBELAB Inc., Seoul, Republic of Korea). The preprocessing steps will include the rejection of channels with an intensity lower than 30 AU, motion artifact correction using temporal derivative distribution repair filtering for optical density data, and the correlation-based signal improvement method for hemoglobin data. Additionally, an oxygen saturation threshold will be set at 5%, and band-pass filtering will be applied at a range of 0.01–0.2 Hz. Resting-state levels of oxyhemoglobin (HbO), deoxyhemoglobin (HbR), and total hemoglobin (HbT; HbO + HbR) will be averaged over a 2 min period across various Brodmann areas of the frontal cortex, including the dorsolateral prefrontal cortex (DLPFC), frontopolar prefrontal cortex (FPC), orbitofrontal cortex (OFC), and ventrolateral prefrontal cortex (VLPFC). The connectivity patterns among these regions will then be analyzed. SCR and ECG data will be analyzed using the “NeuroKit2” package in Python [38]. For SCR data, preprocessing steps will include low-pass filtering to remove high-frequency noise, artifact correction to address distortions, and baseline correction to isolate phasic responses. SCR peaks will be then identified and normalized. The phasic and tonic activity, as well as the amplitude and height of signals, will be extracted. For ECG data, preprocessing steps will include using NeuroKit2 for signal filtering to remove noise, artifact correction to address any distortions, and baseline correction to stabilize the signal. R-peaks will be then detected and intervals such as RR intervals will be computed for further analysis. Heart rate and HRV statistical and spectral analyses will be conducted using the standard methods. Pulse wave signals will be preprocessed by removing noise and deleting signals from sensors where measurement errors have occurred based on visual inspection and then processed into various time-related and frequency-related parameters, such as H1, H3, and H4.

For time-series bio-signals, various machine learning techniques have been shown to be effective in identifying spatiotemporal patterns for predictive and diagnostic tasks [39]. For single-channel signals, such as ECG or SCR, we plan to extract meaningful features that represent the physiological state or detect abnormalities using machine learning models, such as the random forest model. These features include traditional metrics (e.g., R-peak detection and morphological analysis) as well as advanced decomposition techniques (e.g., wavelet transforms and empirical mode decomposition) that facilitate the extraction of time–frequency domain characteristics to capture subtle and non-stationary signal behaviors [40]. Furthermore, deep learning-based methods, including 1D convolutional neural networks (CNNs) and transformer architectures, will be employed for automated and robust representation learning directly from raw signals.

For multi-channel signals, such as fNIRS or pulse wave data, we will utilize both linear and non-linear analytical methods to evaluate signal rhythms and variability. Recently introduced approaches (e.g., network analysis and attention-based models) allow for the modeling of long-term dependencies, revealing spatiotemporal patterns that traditional methods often fail to detect [41,42]. Graph-based models, such as graph neural networks and graph convolutional networks, show potential for analyzing inter-channel connectivity and exploring the functional relationships among multiple bio-signal sources [43]. By leveraging classical connectivity metrics, such as phase-locking values or mutual information, we expect these integrated approaches to yield more accurate and interpretable results.

To address potential confounding variables, we have identified key confounders such as variability in bio-signal measurements, subjectivity in diagnoses, and multi-collinearity among bio-signals. We will use multivariate regression and regularization techniques like Least Absolute Shrinkage and Selection Operator or ridge regression to handle multi-collinearity and apply principal component analysis for dimensionality reduction. Additionally, to ensure our models are based mainly on bio-signals, we will exclude questionnaire scores that strongly correlate with specific patterns. This approach helps maintain model robustness and aligns with our primary study objective.

Additionally, we will investigate local and relative differences in brain activity through amplitude and frequency power analysis. Frequency-domain techniques (e.g., Fast Fourier Transform (FFT), Short Time Fourier Transform (STFT), and wavelet transforms) can be applied to extract spectral features from bio-signals, capturing power distributions across key frequency bands [44]. Recent hybrid approaches transform time-frequency representations into image-like inputs, enabling the use of CNN-based models to automatically discern frequency-related patterns. These methods can be used to enhance the sensitivity of diagnostic tools, particularly for complex and non-stationary bio-signals [45]. The significance and validity of the model will be assessed based on the classification accuracy and the area under the receiver operating characteristic (ROC) curve. These metrics will be obtained through bootstrapping and cross-validation methods and tested against a chance level (0.5, 0.33, or 0.17, depending on the prediction outcomes) using a *t*-test with a significance level of *p* < 0.05.

### 2.9. Participant Safety

All participants will be thoroughly informed of the study’s purpose, procedures, potential risks, and benefits before participation. Written informed consent will be obtained from each participant. Given that the study involves only non-invasive biometric measurements using electrodes and surveys, no adverse reactions are anticipated. However, if a participant experiences any discomfort or adverse events before or during the measurements, the study will be halted immediately. The researcher will monitor the participant’s condition and decide whether to continue the study. If the researcher’s judgment is insufficient, the principal investigator will make the final decision regarding study termination for that participant.

In the event of a serious medical situation during the study, the procedure will be stopped immediately, and appropriate measures will be taken. The incident will be reported to the Institutional Review Board (IRB), and emergency care will be provided at Kyung Hee University Hospital in Gangdong as needed.

## 3. Discussion

This study represents an initial step toward enhancing the objectivity and reliability of pattern identification in patients with FD through the integration of brain–body bio-signals into AI-based diagnostic algorithms. While our current protocol focuses on collecting bio-signals, such as fNIRS, SCR, ECG, and pulse wave under controlled clinical conditions, future advancements are expected to extend beyond these boundaries. Ongoing progress in sensor miniaturization, wearable device technologies, and AI-driven analytics suggests a more integrated and dynamic approach to patient care.

As sensor technology becomes increasingly compact and accessible, it will likely become feasible to gather data outside the clinical setting. For instance, wearable sensors capable of continuously monitoring autonomic activity, subtle variations in pulse waves, or low-level cortical signals can support more naturalistic and longitudinal assessments of patients with FD. By capturing changes in symptoms, lifestyle factors, medication responses, and dietary habits in real-life contexts, these wearable systems may facilitate the early detection of subtle pattern shifts, allowing for timely intervention before symptoms become clinically significant.

The role of advanced AI techniques—such as deep learning and multimodal data fusion—will continue to grow. Integrating signals from multiple physiological domains enables AI models to detect complex patterns that conventional analyses may overlook. Over time, these insights may relate specific brain activities and autonomic responses to FD-associated patterns, thereby refining the conceptual framework of pattern identification in Korean medicine. Such evidence-driven approaches could also contribute to establishing more standardized and reproducible diagnostic criteria, maintaining a balance between traditional clinical expertise and quantitative precision. For instance, integrating brain–body bio-signals with AI algorithms has shown effectiveness in diagnosing subjective pain reports [16,17]. Additionally, when bio-signals are paired with machine learning techniques, they can be utilized to diagnose emotional states such as anxiety [46], pleasure, fear, and anger [47].

In the long term, we anticipate incorporating these technologies into a comprehensive Korean medicine-based health check-up system. By combining time-honored diagnostic insights with state-of-the-art bio-signal analytics, patients could benefit from more personalized and preventive care. Regular assessments—either periodically within clinical environments or continuously via wearable devices—would generate robust data sets, guiding timely interventions, individualized herbal prescriptions, acupuncture treatments, and lifestyle recommendations. Such an initiative could improve patient outcomes, enhance quality of life, and reinforce global confidence in Korean medicine as a scientifically grounded, patient-centered practice.

While using bio-signal measurements as diagnostic tools offers several advantages, there are potential limitations to consider. Firstly, the controlled experimental setting for collecting bio-signal data may not fully capture the variability of symptoms that patients experience in their everyday environments. For instance, FD patients may experience symptoms after meals, which can diminish over time and potentially affect their bio-signals. To address this issue, we will assess the severity of FD symptoms at the time of bio-signal measurement using a Visual Analog Scale (VAS), in addition to the questionnaires. Secondly, despite the integration of multiple bio-signals, accurately correlating these signals with specific FD patterns is inherently complex due to the multifactorial nature of the condition. Multi-collinearity should also be accounted for during data analysis.

In summary, while our current research protocol marks an important step toward quantifying and objectifying FD pattern identification through bio-signals and AI, further developments extend well beyond the present scope. Enhanced sensor technologies, wearable integration, and advanced AI analytics, combined with a holistic Korean medicine health check-up model, hold the potential to transform FD management. This integrated, data-driven strategy may serve as a template for aligning traditional diagnostic principles with cutting-edge scientific methodologies, ultimately offering more nuanced, patient-focused, and effective care.

## Figures and Tables

**Table 1:** Clinical study schedule.

	Visit 1 (Screening)	Visit 2 (Bio-Signal Measurements)
Written consent	●	
Demographic/medical history survey	●	
Vital sign (blood pressure, pulse, temperature) measurements	●	●
Physical measurements (weight, height)	●	
Blood test and electrocardiogram	●	
Visual Analog Scale for dyspepsia/diagnosis of functional dyspepsia (ROME IV)	●	
Confirmation of inclusion/exclusion criteria	●	
Medication history/medication intake survey		●
Questionnaires - Standard Tool for Pattern Identification of FD - Cold–Heat Pattern Questionnaire - Deficiency–Excess Pattern Questionnaire - Phlegm Pattern Questionnaire - Food Retention Questionnaire - Spleen Qi Deficiency Questionnaire - Stomach Qi Deficiency Questionnaire - Nepean Dyspepsia Index - State-Trait Anxiety Inventory - Beck Depression Inventory-II - EeroQqol-5-Dimension		●
Korean medicine diagnosis (interview, tongue diagnosis, pulse diagnosis, abdominal diagnosis)		●
Bio-signal measurements (fNIRS, pulse, SCR, ECG)		●
Adverse events assessment		●
Confirmation of clinical study completion		●

## Data Availability

Data are contained within the article.
